# 

**Published:** 1878-06

**Authors:** 


					﻿Whole Number.
CCCXCVII.
June, 1878.
Number 6.
Vol. XXXVI.
THE
CHICAGO
MEDICAL
J ournal and Examiner
EDITORS:
WILLIAM H. BYFORD, A.M., M.D..
JAS. NEVINS HYDE, A.M., M.D. FERD. C. HOTZ, M.D.
E. FLETCHER INCALS, M.D.
CHICAGO:
PUBLISHED BY THE CHICAGO MEDICAL PRESS ASSOCIATION.
1S8 Clark Street.
2f“Read the Notice of this Journal among Advertisements.
				

